# Editorial: Innovation fermentation empowers the new development of future functional foods

**DOI:** 10.3389/fmicb.2026.2002785

**Published:** 2026-10-07

**Authors:** Hulya Cakmak, Tomás García-Cayuela, Shiqi Li, Jianping Wei

**Affiliations:** 1 Faculty of Tourism, Department of Tourism and Gastronomy Management Programs, Izmir Katip Celebi University, Cigli Izmir, Türkiye; 2 Tecnologico de Monterrey, Escuela de Ingenieria y Ciencias, Zapopan, Jalisco, Mexico; 3 College of Food Science and Engineering, Northwest A&F University, Xianyang, China; 4 College of Food Science and Technology, Northwest University, Xi'an, Shaanxi, China

**Keywords:** bioactive compounds, fermentation, fermented foods, functional foods, microbial community, microbiome engineering, novel fermentation substrates, precision fermentation

Fermentation is a processing method that has long been used for food preservation; however it is a dynamic biotechnological process and fermented foods are actively used in our current diet not only for their organoleptic properties but also dealing with malnutrition, lactose intolerance, and dietary protein deficiencies (Kokisi et al., 2026). The future of fermented foods lies at the intersection of traditional methods and modern science. From beer brewing and sourdough bread to soy sauce, cheese, and yogurt, fermentation has revolutionized food preservation while enhancing flavor profiles and nutritional value.

With growing focus on healthy diets and sustainability, fermented foods are poised to expand from regional to global, from niche to mainstream, and become a vital part of the future food system. Currently, fermentation process and fermented products present both unprecedented opportunities and challenges. This Research Topic would like to showcase cutting-edge innovations in fermentation technology that are driving advancements in next-generation functional foods. Besides, it offers novel insights into the development of fermented foods in the future by combining multidisciplinary research in microbiology, food science, bioengineering, bioinformatics, and other fields. We seek to facilitate their evolution from traditional craftsmanship to precision manufacturing, transform regional specialties into globally accessible products, and ultimately establish them as fundamental components of a healthy and sustainable food system.

Hacifettahoglu and Bilici critically assessed the novel fermented foods in their review. It has been revealed that novel fermented foods represent a significant advancement in sustainable nutrition, offering enhanced digestibility through the breakdown of macronutrients and providing a more diverse gut microbiota. The development of specialized starter cultures and the employment of precision fermentation allow unprecedented control over microbial interactions and the fermentation environment. These biotechnological innovations facilitate the production of foods with better digestibility and enriched micronutrient profiles. However, the authors raise concerns regarding potential biochemical risks and consumer safety. The utilization of genomically modified microorganisms, including those developed through advanced Cas 9-based approaches or metabolic engineering, has sparked debate over regulatory oversight and long-term health implications. Beyond these, the fermentation process can inadvertently lead to the formation of anti-nutrients and allergenic compounds, such as biogenic amines, mycotoxins, and advanced glycation end-products. Moreover, the potential presence of antibiotic resistance genes and pesticide residues in raw materials highlights the risks to be considered in novel fermented foods.

Fermentation is a useful tool for supporting digestibility of food ingredients by enhancing the nutrient bioavailability, reducing glycemic index and eliminating anti-nutritional factors (Hacifettahoglu and Bilici; Cakmak et al., 2025). Alternative protein-rich mopane worm flour and sweet potato flour blends fermented with lactic acid bacteria improve their health benefits by reducing rapidly digestible starch, increased the peak viscosity and reducing gelatinization temperatures which improve their techno-function properties thus be used in special diets including dysphagia (Molimi and Adebo). In order to evaluate the consumption of fermented foods directly on body functions, Wang et al. conducted a research about the effects of kefir-fermented soymilk (SM) consumption on exercise-induced fatigue in mice. They analyzed the biochemical indicators related to fatigue, glycogen metabolism, oxidative stress, gut microbiota composition, and fecal short-chain fatty acids to identify the underlying mechanisms. The muscle and liver glycogen levels of fermented soymilk consuming mice found significantly higher than control groups suggesting that SM may enhance available energy stores during exercise by promoting glycogen synthesis or reducing glycogen consumption, thus delaying fatigue. Besides, SM significantly increased catalase activity and decreased malondialdehyde levels, indicating enhanced antioxidant defense and reduced lipid peroxidation, so reducing exercise-induced oxidative stress.

Fermented grains can be a source of probiotic bacteria. Ravi and Rana stated in their study that fermented pearl millet is a source of acid and bile-tolerant *Bacillus aryabhattai, Pediococcus pentosaceus*, and *Lactiplantibacillus plantarum* which also possess antioxidant activity, and antagonistic activity against foodborne pathogens like *Escherichia coli, Bacillus cereus, and Staphylococcus aureus*. In addition, advancements in whole genome sequencing techniques open a new path for phenotypic characterization of strains responsible for production of traditional fermented products (Huang et al.). Similarly, *Pediococcus pentosaceus* K6 strain isolated from Tibetan kefir grains showed strain-specific probiotic features, strong inhibition against *Listeria monocytogenes*, gastrointestinal tolerance, with reinforced EPS production and biofilm formation capacity that is critical for mucosal adhesion and colonization. Besides, Huang et al. stated that these strains exhibit favorable preliminary safety performance with favorable negative hemolytic activity, absence of harmful metabolites or high risk transferable antibiotic resistance determinants. Besides, Meng et al. investigated the probiotic properties of *Lacticaseibacillus paracasei subsp. paracasei* isolated from Jiangshui (fermented celery, cabbage and wild vegetables) and *Saccharomyces cerevisiae*, along with their functional activities and fermentation performances in *Sonchus oleraceus L*. Jiangshui. The glucose dialysis retardation index and ACE inhibitory activity of *S. oleraceus* were significantly increased after fermentation with probiotics. Also, the content of volatile substances and amino acids related to the flavor of *S. oleraceus* Jiangshui increased significantly after fermentation, and the content of volatile substances in *L. paracasei* and S. *cerevisiae* co-fermented sample. Therefore, these isolated strains with probiotic functions have the potential to be used in novel food formulations enhancing their nutritional composition.

Precision fermentation employing single or multiple strains can provide desirable texture; flavor and color development especially for novel foods including plant-based meats, insect-based blends etc. (Cakmak et al., 2025; Molimi and Adebo). Flavor development is a critical aspect for brewed drinks and spirits especially with complex flavor compositions. In this context, Ren et al. studied the effects of saccharification-promoting *Bacillus velezensis* strains on the volatile profile of fermented grains for production of rice-flavored Baijiu, and further dissected the genetic basis of the key strains. They identified a total of 885 volatile metabolites across all fermented grain samples, with a majority of terpenoids, esters, and heterocyclic compounds, with strong presence of alcohols, hydrocarbons, ketones, aldehydes, aromatics, acids, amines, phenols, nitrogen compounds, and others. The effect of four *Bacillus velezensis* strains on the composition of flavor metabolites during rice flavor Baijiu fermentation was systematically analyzed, and they found that the four strains jointly increased the contents of 304 flavor metabolites, including 78 terpenoids such as trans-geranylgeraniol, (–)-γ-elemene, and α-pinene; 62 esters such as hexanedioic acid dimethyl ester, octanoic acid 2-furanylmethyl ester, and hexanoic acid butyl ester; 49 heterocyclic compounds such as indole and theophylline; and 11 phenolic compounds such as guaiazulene. As a conclusion, FUBL1 and FUBL3 strains showed the most notable enhancement effects, significantly promoting a wider variety of terpenoids and esters and creating a more complex and multi-layered flavor profile with fruity, floral, sweet, and woody notes.

The contributions published in this Research Topic indicate that fermentation science is entering a new period characterized by precision microbial engineering, artificial intelligence, and sustainability-driven innovation. Future progress will depend on interdisciplinary collaboration among microbiologists, food scientists, bioengineers, computational scientists, nutritionists, and industry partners. Such integration will accelerate the development of functional foods that are scientifically validated, environmentally sustainable, and open to evolving consumer health needs and sensory acceptance with hand-in-hand collaboration for regulatory compliance.
